# Insights from the cDNA and EST analysis of Antrodia cinnamomea

**DOI:** 10.6026/97320630017109

**Published:** 2021-01-31

**Authors:** Jing Li, Yanling Liu, Richard Yankey, Dongmei Lin, Zhanxi Lin

**Affiliations:** 1 National Engineering Research Center of JUNCAO Technology, Fujian Agriculture and orestry University, Fujian, Fuzhou 350002, PR China; 2 Life Science College, Fujian Agriculture and Forestry University, Fujian, Fuzhou 350002, PR China

**Keywords:** Antrodia cinnamomea, genome wide analysis, cDNA, EST, GO functional annotation

## Abstract

It is of interest to document the insights gleaned from the cDNA and EST analysis of Antrodia cinnamomea (a fungal species). Hence a library of sequences was constructed and analysed using standard procedures to gain new insights. Therefore, 65 ESTs, with size ranging from 300-2000 bp, were constructed. This included 46 ESTs with definite annotation, 18 ESTs were hypothetical and 1 new protein derived from BLAST analysis. We assigned 227 Gene Ontology terms linked to cell composition, transport, catalytic activity, and regulation functions in these sequences. Moreover, 56 matching genes were found in 8 Kyoto Encyclopedia of Genes and Genomes pathways. Data also showed 271 SSRs from Antrodia cinnamomea ESTs with an occurrence frequency of 96.82%. The STRING data analysis showed 29 genes encoded enzymes highly involved in protein-to-protein interactions linked to expression of regulation function. Thus, we documented some insights from the cDNA and EST analysis of Antrodia cinnamomea for further data mining.

## Background

Antrodia cinnamomea is a rare precious medical fungus found mainly in Taiwan China, which is well known for its immunomodulatory, anti-inflammation and anti-cancer pharmacological attributes [1]. Notably, triterpenoids and sterols are the most classical components and are biosynthesized via the meavalonic acid (MVA) pathway [2]. However, the relative genes and protein involved in the metabolite biosynthesis pathway of A. cinnamomea have rarely been studied. Less molecular information and EST sequences of A. cinnamomea were retrieved in the NCBI database. Complementary DNA (cDNA) library construction application for developing expressed sequence tags (ESTs) and transcriptome, was first reported in 1994 [3]. Gateway technology is a direct approach to speed-up functional open reading frame analysis [4], and the cDNA fragment is directly recombined into the destination vector, which is highly efficient, simplified cloning, chimeric clones reduced, less size bias and easily clone to a large extent, accurately reflecting the expression level of the original abundance of mRNA in the library. Simple sequence repeats (SSRs) are wide range extensive length polymorphisms used in selection of marker-assisted, genetic diversity print-finger, genetic mapping or breeding applications. SSRs are usually made of 1 to 6 repeat nucleotides, which are composed in tandem and are widely distributed in the coding regions and non-coding regions of eukaryotic genes [5]. Screening of EST-SSRs from the cDNA library is simple, inexpensive, and sequence-consistent, hence it is widely used in various plants and fungi such as rice, coffee, beans, rubber tree [6].

## Methodology

### Fungi materials:

A cinnamemea AC001 (Genbank NO: KM925002) was donated by Taiwan Shennong Fungus Biotechnology Co.Ltd. and stored in the National Engineering Research Center of JUNCAO Technology of Fujian Agriculture and Forestry University. The strain was inoculated into a PDA plate and placed at a constant temperature of 28°C, then transferred to a liquid medium [7], shaken at 28°C, 120 rpm in the dark, and cultured for 14, 21, 28, and 35 days. The mycelium was uniformly mixed after 35 days and used to construct a full-length cDNA library.

### Total RNA extraction and integrity assessment:

The total RNA of A. cinnamomea was extracted by TRIzol method [8], and the total RNA integrity and quality analysis detected by electrophoresis on a 1% agarose gel and Nanodrop2000C (Thermo, USA). The mRNA was isolated by the FastTrack MAG Maxi mRNA Isolation Kit (Thermo, USA). cDNA synthesis was performed by the CloneMiner II cDNA Library Construction Kit (Thermo, USA).

### Full-length cDNA library construction:

The cDNA library of A. cinnamomea was constructed by using the Gateway method [9]. Briefly, the cDNA was used as a template to synthesize the first strand and the second strand of cDNA. The cDNA obtained by transcription was linked to three different reading frames of the adaptor, and Homologous Recombination performed the recombinant method. The primary cDNA library was prepared by cloning into the pDONR222 vector. Then, the primary cDNA library mixed plasmid was extracted, and a yeast two-hybrid cDNA library (secondary library) was prepared by LR recombination into the yeast vector pGADT7-DEST. Library identification and determination. The primary and secondary libraries of the obtained cDNA were diluted in a ratio of 1:1000 and coated with a plate. After incubating for 10 h at 37°C, single colonies were observed and counted. The titer of the amplified library was calculated as follows: (number of plaques x dilution factor x 103 µL/mL)/(diluted phage plated µL). The dilution factor is 1 x 104. The inserted sequences were randomly selected for PCR amplification using pDONR222-F primers: 5'-TCCCAGTCACGACGTTGTAAAACGACGGCCAGTCTT-3', pDONR222-R primers: 5'-AGAGCTGCCAGGAAACAGCTATGAC CATGTAATACGACTC-3'. The total volume of PCR was 20 µL, containing 1 µL template, 10 µL 2xPCR Master Mix (Thermo, USA), 1 µL each primer and 7 µL ddH2O. Cycling conditions were as follows: 94°C for 5 min, followed by 29 cycles of 95°C for 30 s, 57°C for 40 s and 72°C for 60 s, followed by 72°C for 5 min.

### Bioinformatic analysis:

The clone transformants were randomly selected and sequenced by Bio-Sun Biotechnology Co., Ltd (Fujian, China). The adapter sequences were trimmed by Vecscreen tool (www.ncbi.nlm.nih.gov/tools/vecscreen) of National Center for Biotechnology Information (NCBI) and the inaccurate bases, poly-A tails, low-quality sequences (<100 bp) and other fragments [10] by CAP3 software removed to finally obtain more accurate sequencing. The NCBI BLAST program was used to compare the spliced single copy sequence with the nucleic acid library (NT) and protein (NR) databases (www.ncbi.nlm.nih.gov/) to complete the homology alignment of BLASTX. The database results with E value <1x10-5 were generally regarded as a significant match [11]. NCBI's non-redundant protein database followed by the assignment of functionality via Gene Ontologies (GO) [12] using BLAST2GO (www.blast2go.com/) [13]. The GO analysis included functional classification of Molecular Function, Cellular Component, and Biological Process for EST sequence data by BLAST2GO software. Pathway assignments were mapped according to Kyoto Encyclopedia of Genes and Genomes (KEGG) database, while interaction protein and nucleic databases was conducted for biological functions by the STRING network (www. string-db.org/).

### EST-SSR analysis:

Screening of EST-SSR was performed using the GRAMENE SSR online tool (https://archive.gramene.org/db/markers/ssrtool) withthe standard length for searching for SSR including dinucleotide, trinucleotide, tetranucleotide, pentanucleotide, hexanucleotide with a minimum number of repetitions of 7, 5, 4, 4, 3. The SSR frequency and length were statistically analyzed.

## Results and Discussion:

### Total RNA assessment:

**Figure 1:**
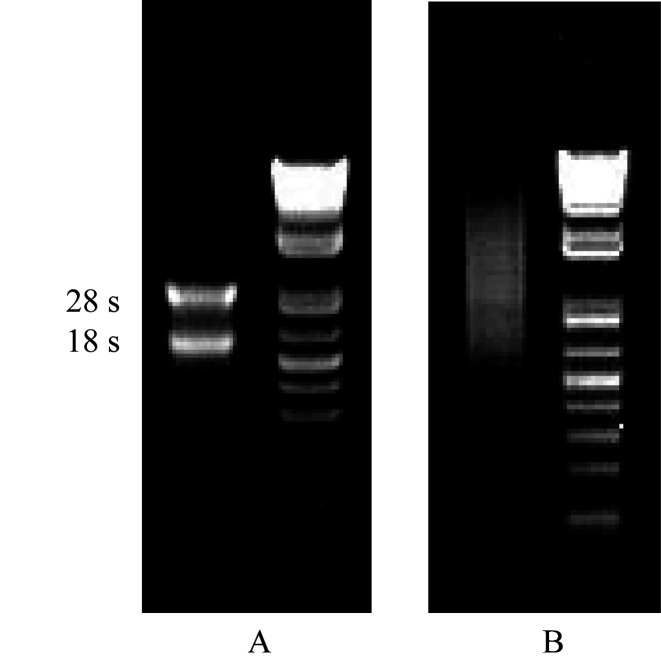
Electrophoresis of total RNA and mRNA of A. cinnamomea mycelium.

The total RNA extracted from the mycelium of A. cinnamomea was detected to be 963 ng/µL, the total amount was 481 µg, and the OD260/OD280 was 2.14, which indicated that the total RNA was integrated and stable enough for cDNA library construction. The integrity was measured by 1% agarose gel electrophoresis as shown in Figure 1A, and mRNA was further isolated by total RNA isolation (Figure 1B).

### Construciton of cDNA library:

**Figure 2:**
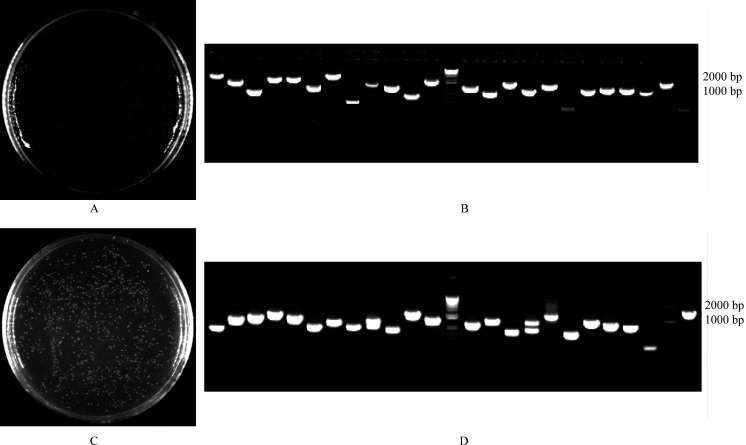
Identity and length analysis of primary (A, B) and secondary cDNA library (C, D).

The full-length cDNA primary library was transformed into a plate dilution of 1:1000, the library titer was 5.2 x 106 CFU/mL, and the total number of clones was 1.0×107 CFU (Figure 2A). The secondary library recombination rate was as high as 95%. The transformation plate dilution is 1:1000, the library titer is 6.1x106 CFU/mL, Total number of clones is 1.2x107 CFU (Figure 2C). The clones were selected randomly and amplified by PCR using pDONR222-F and pDONR222-R primers. The PCR products were detected by 1% agarose gel electrophoresis and the recombinant rate was 95%. The insert size is 300-2000 bp, and the average length is 1000 bp (Figure 2B and D), indicating that the library contains relatively long cDNAs that meet library capacity requirement. This method directly used cDNA as a template to synthesize cDNA double strands without any amplification process, and effectively kept the genetic information of full-length gene function. It also afforded an important resource for information about genes at the transcriptional level. The fragment is long enough to reflect the natural structure of the gene as much as possible, thus making it easier to obtain the complete sequence and functional information of the target gene in the library [14] and 95% of the recombinant cDNA library indicating the quality and providing a basis of the depth of the study.

**Figure 3:**
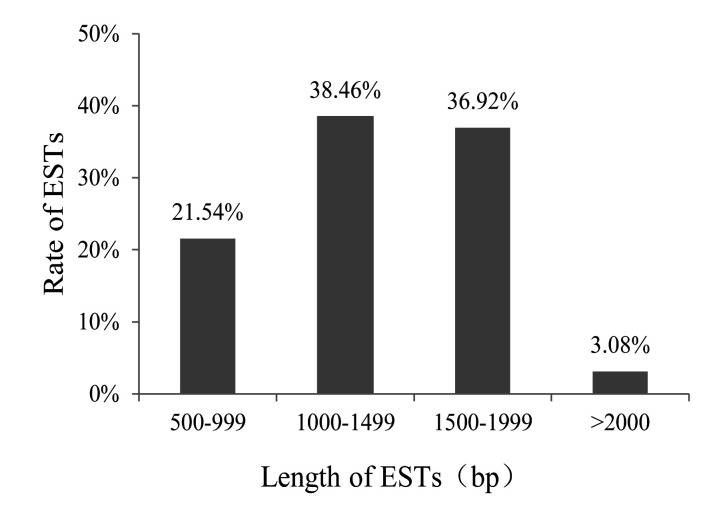
Distribution of ESTs lengths.

Several clones were randomly picked from the cDNA library for sequencing, and a total of 65 valid single sequences (unigene) were obtained after removing the vector sequence and low-quality sequences (<100 bp). As shown in Figure 3, 14 ESTs length were 500-999 bp, 21 ESTs length were 1000-1499 bp, 24 ESTs length were 1500-1999 bp and 2 ESTs length were >2000 bp. Among them, the longest EST was 3474 bp, and the shortest EST was 573 bp. The CAP3 program was spliced to obtain a unique sequence consisting of one contigs and 64 single singlets. A total of 65 individual ESTs were analyzed and submitted in Genbank (Table 1 - see PDF). The spliced sequence was subjected to homology alignment and gene function annotation with the NR database using NCBI BLASTX program. 65 single sequences except one (1.54%) had no significant homology; the remaining 64 (98.46%) had significant homologous sequences, including 46 known functional proteins and 18 unknown proteins. The homologous proteins were observed in Taiwanofungus camphoratus, Grifola frondosa, Wolfiporia cocos and fibroporia radiculosa etc. Obviously, 1 (Genbank NO.: MN205389) of the ESTs of the constructed library was noted to be novel and uncharacterized, and putatively expressed proteins with no homology matches of any public databases. Thus, its functional characterization and possible role in A. cinnamomea will be worthwhile to investigate.

### GO annotation analysis:

The single sequence of functional annotations was classified into functional categories in BLAST2GO software. A total of 227 GO terms were obtained, with an average of 6.837, divided into 34.36% biological processes, 27.75% cellular components, and 37.88% molecular functions, with the distribution of the ESTs (Suplementary Figure 1 - see PDF). According to the biological process, the component contained metabolic process (37%), cellular process (31%), biological regulation (9%), localization (8%), cellular component organization or biogenesis (8%) and regulation of biological process (7%), as shown in Supplementary Figure 1A (see PDF). Regarding molecular function, 53% ESTs were associated with catalytic activity, 38% ESTs were associated with binding and only 8% ESTs were associated with transporter activity (Supplementary Figure 1B see PDF). The components of biological cellular process associated with the ESTs included membrane (19%), membrane part (17%), cell (16%), cell part (16%), organelle (13%), organelle part (8%), protein-containing complex (6%) and membrane-enclosed lumen (4%) (Supplementary Figure 1C - see PDF). Supplementary Figure 2A and 3A shows the enrichment of gene functions. There are 5 GO terms in biological process that have highly enrichment value including cellular process (GO:0009987), phosphorylation (GO:0016310), cellular metabolic (GO:0044237), metabolic process (GO:0008152), oxidation-reduction process (GO:0055114), with 27, 6, 21, 32, 10 background genes respectively. According to molecular function GO term, there is catalytic activity (GO: 0003824) and oxidoreductase activity (GO:0016491) significantly enriched with 32 and 10 background genes (Supplementary Figure 2B and 3B - see PDF). In cellular component GO term, cell nodescore (GO: 0005623), membrane nodescore (GO:0016020), intracellular part (GO: 0044424), integral component (GO:0016021), with 22, 26, 21 and 23 backgroud genes, respectively (Supplementary Figure 2C and 3C - see PDF).

### KEGG pathway and Interaction protein analysis:

**Figure 4:**
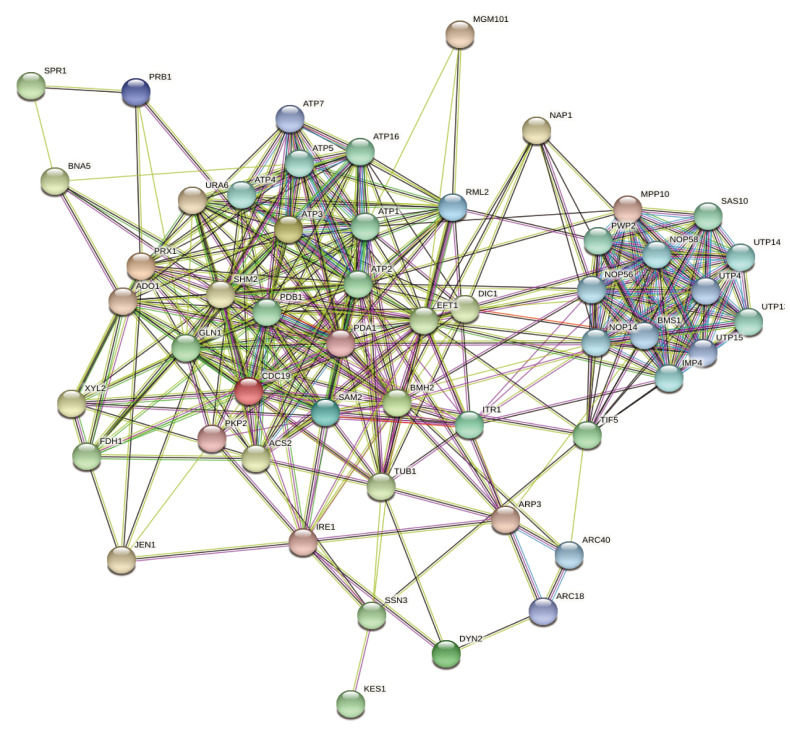
Network nodes represent proteins: experimentally determined (purple), gene neighborhood (green), gene fusions (red), gene co-occurrence (blue), co-expression (black).

KEGG is an approach to link genomic data with higher order functional sequence by computerizing current information on cellular processes and by standardizing gene annotations. It provides biochemical pathways for the annotations species in which the genome have been discovered. According to Table 2 (see PDF), a total of 56 matching proteins were revealed to be involved in 8 KEGG pathways. 19 of 65 ESTs were annotated to the metabolic pathways (sce01100), which are the most represented pathways. While, the ribosome biogenesis in eukaryotes and oxidative phosphorylation are the second and third most represented pathways. In addition, carbon metabolism, pyruvate metabolism, glycolysis gluconeogenesis, methane metabolism and glycosylate and dicarboxylate metabolism were also represented. The interaction among the 65 ESTs were analysed by STRING database with the genome of Sachharomyces cerevisiae. The result showed a functional association network determined with 53 codes, 346 edges and PPI enrichment p-value of 0.00131 (Supplementary Table 1 see linked excel file). The 65 ESTs with the low confidence (0.150) minimum required interaction score is no more than 20 interactors. The predicted potential regulators with no clustering are shown in Figure 4. The STRING results revealed protein ATP2, PDB1, ATP1, ATP16, SAS10, PWP2, UTP13, ATP5, ATP4, UTP14, NOP58, NOP14, NOP56, ARC40, BMS1, UTP4, UTP15, ATP7, ARC18 were the functional partners. As Supplementary Figure 1 (see PDF) shows, there are a total of 11 GO terms which have high enrichment value including cellular process, phosphorylation, cellular metabolic, metabolic process, oxidation-reduction process, catalytic activity and oxidoreductase acitivity, cell nodescore, membrane nodescore, intracellular part, integral component of the A. cinnamomea cDNA library. In the present study, the STRING for A. cinnamomea is primarily based on the Sachharomyces cerevisiae genome. We sequenced among the matches, 29 genes which encoded enzymes highly involved in Ribosome biogenesis in eukaryotes, oxidative phosphorylation, carbon metabolism, glycolysis/ gluconeogenesis, glyoxylate and dicarboxylate metabolism, pyruvate metabolism and methane metabolism (Table 2 - see PDF).

### EST-SSR analysis:

A total of 271 SSRs were isolated from 63 ESTs of A.cinnamomea with an SSR occurrence frequency of 96.82%. Among the EST-SSRs, there are 7 ESTs with 1 SSR, 12 ESTs with 2 SSRs, and 44 ESTs with 3 or more. The tri-nucleotides results shown in Table 3 (see PDF) accounts for the largest proportion of all SSRs. The di-nucleotide and tri-nucleotide repeat SSRs in the EST accounted for 95.31% of the total repeats, and the number of dinucleotide repeat motifs GC, CG, CT, and TC appeared more frequently with repeats of 14.76%, 13.28%, 9.59%, and 9.22%, respectively (Table 4 - see PDF). However, AG, GT, and TA were less frequent motifs accounting for less than 5%. In the tri-nucleotide repeat motif, AGG, GAA and GTC appear slightly higher, accounting for 3.33%, while CAC, CGC, AGC and ATC only occur once. The frequencies of tetra-nucleotides, hexa-nucleotides and hepta-nucleotides are all very low. However, di-nucleotide SSRs (18.75%) and tri-nucleotide SSRs (76.56%) presented higher polymorphic proportions than tetra- nucleotide in A. cinnamomea, which suggested that the SSRs which occurred within untranslated region were more polymorphic than those in exon regions.

## Conclusion

In this study, we document some insights from the cDNA and EST analysis of Antrodia cinnamomea for further data mining. This is helpful in the genome level understanding of Antrodia cinnamomea for applciation in biomedicine.
